# A Bayesian Framework for Parameter Estimation in Dynamical Models

**DOI:** 10.1371/journal.pone.0019616

**Published:** 2011-05-24

**Authors:** Flávio Codeço Coelho, Cláudia Torres Codeço, M. Gabriela M. Gomes

**Affiliations:** 1 Instituto Gulbenkian de Ciência, Oeiras, Portugal; 2 Escola de Matemática Aplicada, Fundação Getúlio Vargas, Rio de Janeiro, Brazil; 3 Programa de Computação Científica, Fundação Oswaldo Cruz, Rio de Janeiro, Rio de Janeiro, Brazil; Albert Einstein College of Medicine, United States of America

## Abstract

Mathematical models in biology are powerful tools for the study and exploration of complex dynamics. Nevertheless, bringing theoretical results to an agreement with experimental observations involves acknowledging a great deal of uncertainty intrinsic to our theoretical representation of a real system. Proper handling of such uncertainties is key to the successful usage of models to predict experimental or field observations. This problem has been addressed over the years by many tools for model calibration and parameter estimation. In this article we present a general framework for uncertainty analysis and parameter estimation that is designed to handle uncertainties associated with the modeling of dynamic biological systems while remaining agnostic as to the type of model used. We apply the framework to fit an SIR-like influenza transmission model to 7 years of incidence data in three European countries: Belgium, the Netherlands and Portugal.

## Introduction

Mathematical models have long played a key role in understanding infectious disease epidemiology [1] as well as other biological dynamical systems. Their ability to combine established theory and data to predict empirical observation is unique and cannot be easily achieved by other methods [2]. In such models, data in the form of rate parameters and time-series, and theory in the form of the model formulation, interact to provide insight about each other. Parameter estimation and model selection techniques allow us to improve theory with the help of data (model selection) and estimate data which cannot be directly observed, with the help of theory (parameter estimation).

Proper representation of the intrinsic uncertainty associated with dynamic models of biological systems has been under increasing scrutiny through the development of a number of methods for parameter estimation and model calibration [3]–[10]. Such methods, to be effective, must strive to be as comprehensible as possible in the treatment of all identifiable sources of uncertainty related to a given mathematical representation of a biological system [5]. In practice, however, many uncertainty analysis methods fall short of this ideal. Some of the work in the recent literature focus on developing exact methods for parameter estimation, requiring, for instance, the derivation of the full likelihood function for the model at hand. Exact methods, however, tend to be closely coupled to a specific model or class of models, being less generally applicable [11]–[14].

In this paper we introduce a Bayesian framework for parameter estimation in dynamic models that is applicable to both deterministic and stochastic models [15]. The framework extends similar frameworks proposed for different types of models [4], [6], [16], [17] and focuses of the analysis of dynamic models where full or partial time-series data are available for the model to be fit against. The fitting process estimates the posterior probability distributions for both the model's parameters and output series.

To ensure generality, the dynamic model, from the point of view of the inference machinery, is treated as a “black box” with inputs (parameters) and outputs (time-series), and the full uncertainty about each of these elements can be included in the form of prior distributions which will get updated based on observational data. Model comparison and selection analyses are facilitated by the pluggable nature of the model in the framework.

To illustrate the use of this framework, seven-years long time-series of influenza-like illness incidence data from Belgium, Netherlands and Portugal [18] were used to as a basis for parameter estimation of a deterministic influenza transmission model.

## Methods

The core of the analytical framework proposed was inspired on the Bayesian Melding method [6] with modifications to make it work with dynamic models, that is, with time-series as model outputs. The Bayesian Melding method pioneered in providing a formal inferential framework that took into full account information available about a model's inputs and outputs. We proceed to give a brief description of the Melding method. For a complete description, see the original work. Let 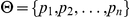 be the set of 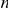 parameters which are the inputs to the model 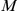. The 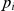 are random variables with a joint probability prior distribution denoted by 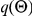. Therefore, 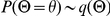. Also let 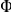 be the set of 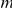 outputs of 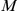, 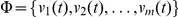.

Since 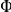 is a function of 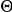, the prior distribution of 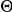, 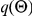 induces a prior probability on 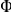, 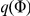:
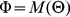
(1)

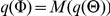



Let 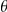 and 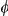 be realizations of the model's inputs and outputs, respectively, such that 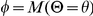. The inferential problem consists in finding the joint posterior probability distribution of 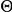, 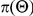, and that of 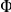, 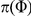, given existing data (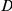). Data will enter the inference in the form of time-series corresponding to the models outputs. Data on the model's parameters can also be used to update 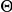
's joint prior probability distribution. The observed data used to fit the model may refer to only a subset of the model's outputs (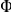). The likelihood of the model's outputs is given by:
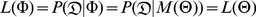
(2)


From equation 2, we see that data on the outputs will inform the likelihood of both 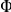 and 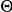 as they are connected by the model. In practice this means that the most likely sets of parameters (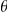) will be the ones which generated the most likely outputs (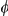). The dependency of the outputs on inputs is given by the model so the accuracy of the inference will depend of the model's identifiability, i.e. different 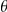 generate different 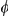.

The posterior of 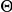 is updated according to equation 3.
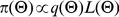
(3)


As already mentioned, this work introduces some extensions to the original Melding method. A couple of extensions stand out. One of them is the ability to use time-series data, the Bayesian Melding method made inferences based on data on single point in time. The second was the use of a multi-chain Markov-chain sampler to more efficiently tackle non-convex higher dimensional parameter-spaces.

### Prior Information

Before starting the inference, prior probability distributions for the parameters in 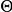, 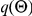, must be defined. The initial conditions for the model can be fixed or included as members of 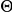. If prior information about the distribution of the outputs is available, it can be pooled with the induced prior on the outputs as described by Poole and Raftery [6]. In the particular application described below, we have used uninformative priors – 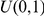 – for the outputs of the models since we had no expectations about them which could inform different prior distributions.

### Likelihood Calculations

The exploration of the parameter space is done by Markov Chain Monte Carlo, as described below until 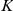 samples are accepted. For the application presented here, the error distribution of 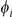, where 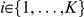, is assumed to be Normal, 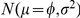. Thus 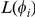 is a Normal likelihood function with fixed variance 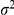. Other parametric forms for the likelihood function can be adopted. Parameters values (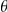) are retained with probability proportional to the likelihood of 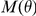, as given by:
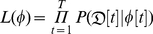
(4)


### Monte Carlo Simulations

A multi-chain differential evolution adaptive metropolis algorithm (DREAM) [19] was used to sample the joint posterior probability distribution of 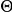, 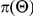. DREAM is a sophisticated algorithm where multiple adaptive chains are run in parallel with delayed rejection.

For the application presented, 16 chains (same as the dimensionality of the parameter space) were started from 16 randomly chosen points in parameter space and moved around with steps given by a gaussian proposal distribution centered at its current position with covariance being adapted every ten steps as described by Andrieu and Thoms [20]. Proposed 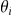 are accepted proportionally to their posterior probability. The chains are run until the desired number of samples is reached after discarding a pre-determined number of burn-in samples. Convergence of the parallel chains was verified at every 100 iterations by the calculation of the Gelman-Rubins' R convergence diagnostic [21].

### Application to Multi-Season Influenza Transmission

We used a deterministic model for influenza transmission, adapted from the Susceptible-Infected-Recovered (SIR) framework [1], to explain multi-season dynamics of influenza in Europe. The model was fitted to two sets of influenza-like illness incidence times-series (Influenzanet [18] and EISN [22]) collected between from 2004 and 2010 in Belgium, Netherlands and Portugal. The model differs from the standard SIR in that only a fraction, 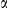, of the infected individuals is symptomatic and infectious, the remaining being asymptomatic and ineffective in passing on the virus. A small infectious immigration rate (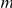) is also added. The model is implemented as a set of ordinary differential equations:
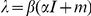


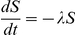


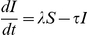


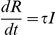
where the recovery rate (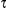) is such that the infectious period last 5 days [23], and the migration parameter (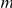) is assumed to be proportional to the number of susceptibles, considering that infection is imported by susceptible individuals who acquire the virus while traveling abroad.

To model the seasonality of influenza epidemics in Europe, the transmission rate 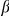 is assumed to drop during the three summer months (June, July and August), thus virtually interrupting transmission of the disease, possibly due to school closure for summer vacations. For the rest of the year 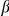 is assumed to be large enough to allow for sustained transmission. During this period the effective reproduction number, 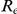, is given by the expression:
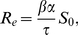
(5)where 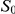 is the number of susceptibles at the beginning of each transmission season.

The model is parameterized in such a way that total population is normalized to 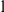 and 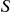, 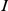, and 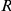 are fractions of the total population. The initial fraction of susceptibles, 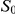, was estimated along with other parameters of the model for each year while the initial fraction infected was set to match the prevalence of the first week of data. The remainder of the population was placed in the 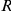 compartment. The symptomatic fraction of 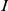, denoted by 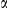, was also estimated for each year. The output of the model, as represented by 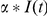 was fitted against the data.

For each country, we have estimated 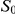 and 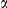 as season specific parameters, while 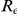 and 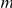 where fixed across the multiple seasons. From these 16 estimated quantities, 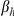 can be calculated by manipulating expression 5 if desired.

The model was fitted to the three countries' datasets. Uniform priors were attributed to all parameters: 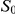 had 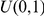 priors for all years; 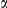 had 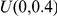 priors for all years; 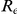 had 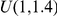 and 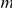, 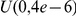. The posterior distribution for parameters and series were obtained from 2000 samples generated by the DREAM algorithm after 2000 burn-in samples were discarded.

## Results and Discussion

**Figure 1:**
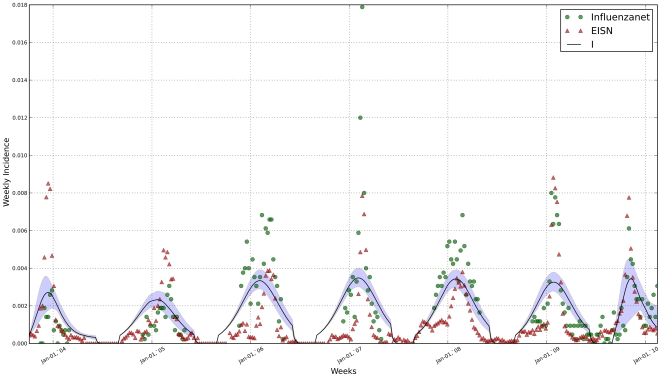
Belgian incidence data and model fit. Incidence median curve (black line) and 95% credible intervals (shaded area) for the model-generated incidence series. The model was fitted simultaneously to Influenzanet data (green circles) and EISN data (red triangles).

**Figure 2:**
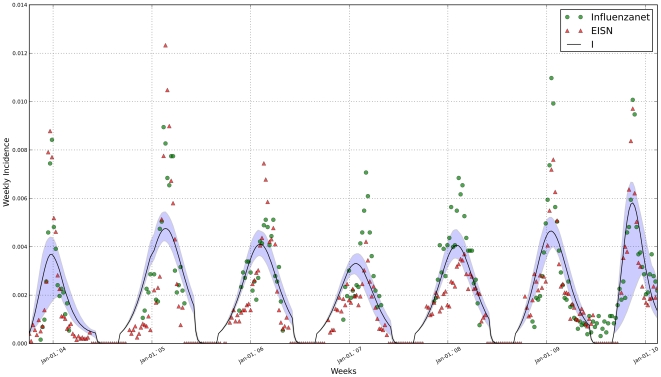
Incidence data from the netherlands and model fit. Incidence median curve (black line) and 95% credible intervals (shaded area) for the model-generated incidence series. The model was fitted simultaneously to Influenzanet data (green circles) and EISN data (red triangles).

**Figure 3:**
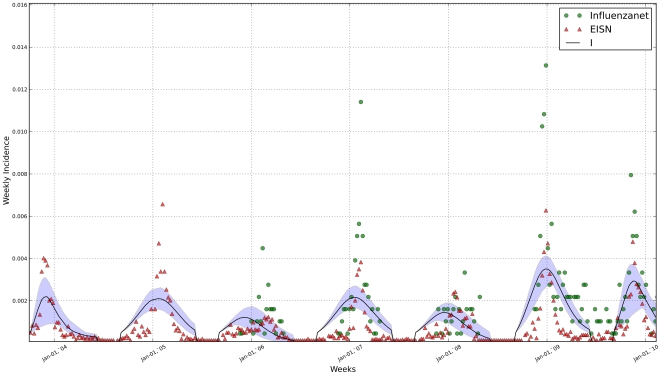
Portuguese incidence data and model fit. Incidence median curve (black line) and 95% credible intervals (shaded area) for the model-generated incidence series. The model was fitted simultaneously to Influenzanet data (green circles) and EISN data (red triangles).

Figures 1, 2 and 3 show the fit of the model against data from both Influenzanet and EISN for the three countries. The model was able attain a good fit to the data, allowing for reasonably precise estimate of the parameters (table 1). We have performed some consistency checks on the estimates obtained (not shown). In particular we have found a positive correlation between the fraction of infections that are symptomatic in a given season (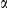) and the time of the epidemic peak (measured from September 1st), suggesting a role of weather factors in the performance of influenza surveillance systems, which is further explored in van Noort *et al.* [24] by combining data from other sources. Although here we chose the simplest model formulation for the purpose of illustration of the parameter estimation method, the results are compatible with other studies. Moreover, the procedure is readily applicable to more elaborate models.

**Table 1:** Model Parameters; posterior estimates.

Name	Belgium	Netherlands	Portugal
	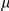 ( 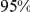 interval)	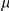 ( 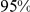 interval)	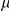 ( 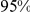 interval)
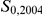	0.246 (0.202, 0.49)	0.337 (0.245, 0.5)	0.215 (0.126, 0.498)
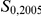	0.434 (0.302, 0.562)	0.805 (0.454, 0.93)	0.493 (0.363, 0.639)
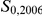	0.644 (0.453, 0.766)	0.685 (0.411, 0.815)	0.265 (0.122, 0.5)
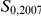	0.669 (0.423, 0.77)	0.543 (0.346, 0.657)	0.519 (0.374, 0.66)
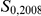	0.645 (0.404, 0.775)	0.67 (0.385, 0.789)	0.316 (0.144, 0.5)
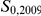	0.588 (0.416, 0.699)	0.664 (0.435, 0.764)	0.577 (0.43, 0.707)
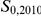	0.299 (0.205, 0.523)	0.43 (0.326, 0.592)	0.336 (0.222, 0.609)
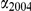	0.0186 (0.00148, 0.0901)	0.0776 (0.0032, 0.332)	0.152 (0.00227, 0.481)
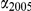	0.258 (0.0768, 0.394)	0.279 (0.12, 0.395)	0.236 (0.0689, 0.396)
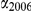	0.306 (0.0972, 0.444)	0.271 (0.112, 0.393)	0.245 (0.0409, 0.396)
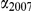	0.278 (0.116, 0.395)	0.301 (0.108, 0.398)	0.263 (0.0893, 0.391)
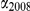	0.228 (0.0447, 0.387)	0.258 (0.0909, 0.39)	0.249 (0.0698, 0.392)
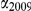	0.152 (0.0426, 0.289)	0.088 (0.012, 0.278)	0.0591 (0.0136, 0.259)
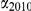	0.107 (0.000647, 0.484)	0.0647 (0.00127, 0.424)	0.0929 (0.00248, 0.46)
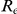	1.1(1.09, 1.16)	1.11, (1.1, 1.18)	1.08, (1.06, 1.15)
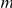	1.78E-06 (1.35E-07, 2.95E-06)	1.98E-06 (1.05E-07, 2.97E-06)	2.84E-06 (8.98E-07, 3.92E-06)
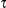	1.4	1.4	1.4

Parameters of the SIR model. Single numbers are values of fixed parameters. The rest are posterior means and their 95% band. 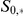 are the initial fraction of susceptibles at each year; 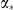 are the fraction of symptomatics for each year; 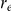 is the effective reproductive number at the beginning of the season; 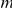 is the infectious immigration constant; 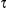 is the recovery rate.

The estimates of the basic reproductive number (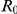) for each season and country, can be obtained by dividing the 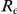 estimated for each country by the 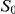 estimated for each year (table 1). Its values range from 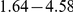 for Belgium, 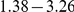 for the Netherlands, and 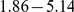 for Portugal. These values, are in accordance to previously reported estimates of 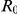 for influenza [25]–[27].

This work proposes a methodological framework to perform parameter estimation in dynamical models where time series data is available for the model to be fit against. The method described can be applied to a wide range of dynamical models, taking its utility beyond the application described in this paper. Currently, its applicability is limited in practice by the robustness of the MCMC samplers available in handling complex high-dimensional parametric spaces. This limitation can be reduced in the future by the development of more powerful posterior sampling methods.

The pluggable nature of the model, in the framework, allows for a simple way to compare multiple models and select which one fits best the available data. Goodness of fit statistics such as AIC [28], BIC [29] or DIC [30], provided by the framework, can be used for this. Model comparison and selection techniques are, however, not discussed in this paper but can be found in the literature [31].

For this work, an open-source software library [32] was developed which allows for the immediate application of the framework proposed here to other models by means of a simple Python script (as decribed in the library's documentation). The library can also be used from within a Sage worksheet [33], requiring little programming knowledge.
